# Infarto Agudo do Miocárdio com Trombose Coronária em um Paciente com Covid-19 sem Fatores de Risco para Doença Cardiovascular

**DOI:** 10.36660/abc.20200972

**Published:** 2021-03-03

**Authors:** Tainá Viana, Mariana Lins Baptista Guedes Bezerra, Rodrigo Morel Vieira de Melo, Cristiano Guedes Bezerra, Vítor Mamédio, Gabriela Pio Dourado, Clara Salles Figueiredo, Luiz Carlos Santana Passos

**Affiliations:** 1 Universidade Federal da Bahia SalvadorBA Brasil Universidade Federal da Bahia, Salvador, BA - Brasil; 2 Hospital Ana Nery SalvadorBA Brasil Hospital Ana Nery, Salvador, BA - Brasil

**Keywords:** Infarto do Miocárdio, COVID-19, Betacoronavírus, Adulto Jovem, Trombose Coronária, Terapia Trombolítica

## Relato de Caso

Homem jovem de 32 anos, sem fatores de risco cardiovasculares, procurou uma Unidade de Emergência apresentando quadro de dor torácica intensa há 30 minutos da admissão, sem irradiação. Sinais vitais admissionais: temperatura corporal de 36,1 ^o^C, frequência cardíaca de 89 bpm e saturação de O_2_ de 96% em ar ambiente. Durante o interrogatório sistemático, o paciente relatou quadro de anosmia e ageusia há dois dias e negou sentir febre ou qualquer outro sintoma respiratório. Paciente se apresentava previamente hígido, negou uso de drogas ilícitas ou ter histórico pregresso de angina. Negou também histórico familiar de infarto agudo do miocárdio ou de doença arterial coronariana.

**Figura 1:**
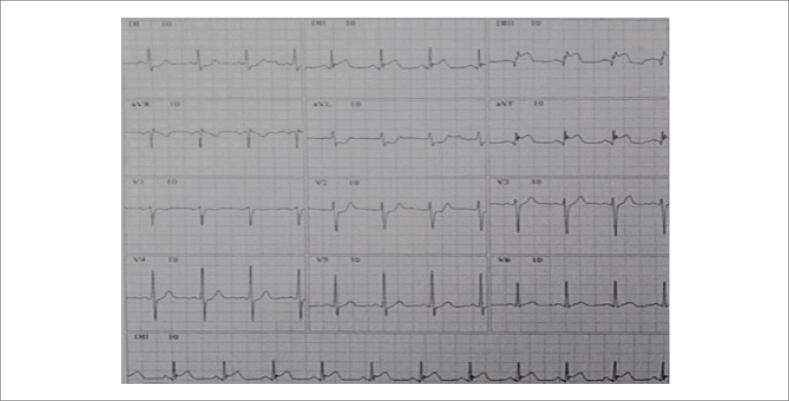
Eletrocardiograma admissional: Eletrocardiograma apresentando supradesnivelamento do segmento ST nas derivações DII, DIII e aVF e infradesnivelamento do segmento ST em DI e aVL, compatível com infarto agudo do miocárdio de parede inferior.

O eletrocardiograma (ECG) de 12 derivações apresentou elevação do segmento ST em DII, DIII e aVF; e infradesnivelamento em DI e aVL, compatível com infarto agudo do miocárdio acrescido de supradesnivelamento do segmento ST IAMCSST de parede inferior (Figura 1) e troponina positiva. Outros exames laboratoriais mostraram alteração da proteína C reativa de 6,7 mg/L (VR: <10 mg/L), ferritina 350,2 ng/mL (VR: 21,8 a 274,6 ng/mL), LDH 5.600 U/L (VR: 120 a 246 U/L) e leucocitose de 12.450 cel/uL. A troponina, ultrassensível, estava acima de 50 ng/mL (VR: <0,034 ng/mL).

**Figura 2:**
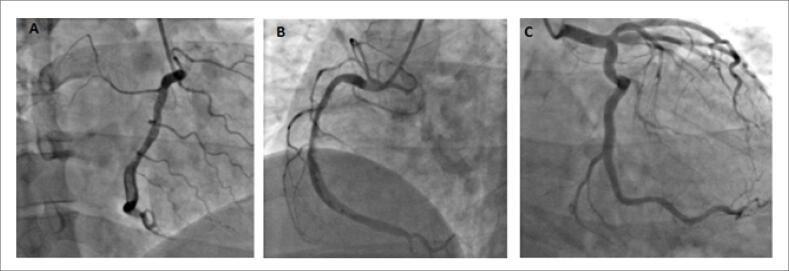
Angiografia coronariana: (A) Coronária direita com uma grande quantidade de trombo em suas porções medial e (B) distal. (C) Coronária esquerda sem lesões ateroscleróticas.

O paciente recebeu uma dose de ataque de dupla terapia antiplaquetária (AAS 300 mg e clopidogrel 300 mg). Como não seria possível seu encaminhamento para angioplastia em menos de 120 minutos, optou-se por terapia fibrinolítica, utilizado tenecteplase (delta T de 5 horas e 36 minutos). O ECG pós-trombólise mostrou redução de 50% da elevação do segmento ST, porém o paciente ainda apresentava dor torácica. Considerada a falha de reperfusão, o paciente foi encaminhado para a angioplastia de resgate. A cineangiocoronariografia (Figura 2; Vídeos 1 e 2) revelou artéria coronária direita com grande quantidade de trombo em segmento médio e distal, sem lesões obstrutivas ateroscleróticas em outras coronárias.

**Vídeo 1:**
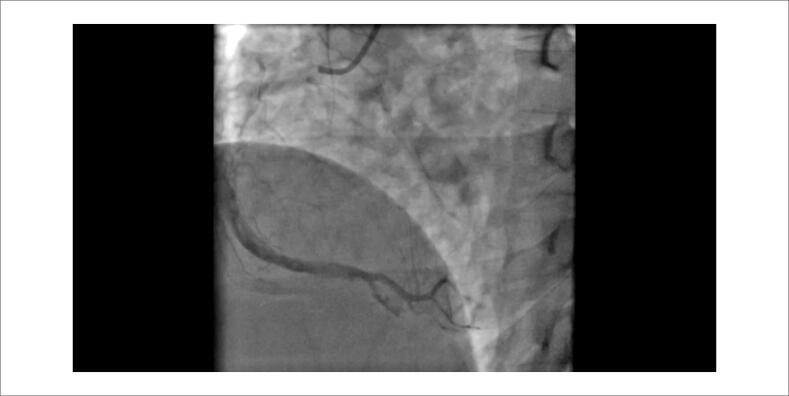
Angiografia da coronária direita: A angiocoronariografia apresenta uma coronária direita com uma grande quantidade de trombo em suas porções medial e distal.

*Link: http://abccardiol.org/supplementary-material/2021/11603/2020-0972-Video01.mp4*

**Vídeo 2:**
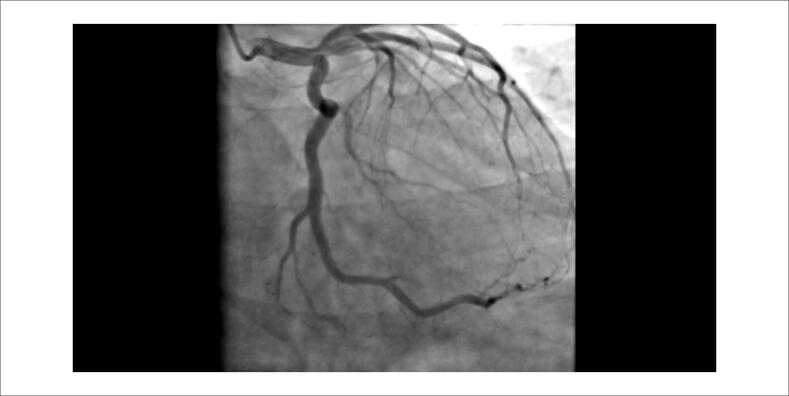
Angiografia da coronária esquerda: A angiocoronariografia não apresenta evidência de lesão obstrutiva aterosclerótica nas demais coronárias.

*Link: http://abccardiol.org/supplementary-material/2021/11603/2020-0972-Video02.mp4*

Devido à alta carga trombótica, apesar da redução do fluxo (TIMI 2), optou-se por não intervir e pelo uso de dupla terapia antiplaquetária associada a heparina de baixo peso molecular em dose terapêutica por 72 horas. O paciente foi transferido para a Unidade de Terapia Intensiva com alívio completo dos sintomas. Devido a suspeita de covid-19, realizou-se pesquisa com *swab* nasofaríngeo por RT-PCR para SARS-CoV-2, cujo resultado foi positivo. A tomografia computadorizada de tórax não mostrou alterações. Nenhuma terapia específica foi instituída para covid-19, pois o paciente permaneceu sem sintomas respiratórios.

**Figura 3:**
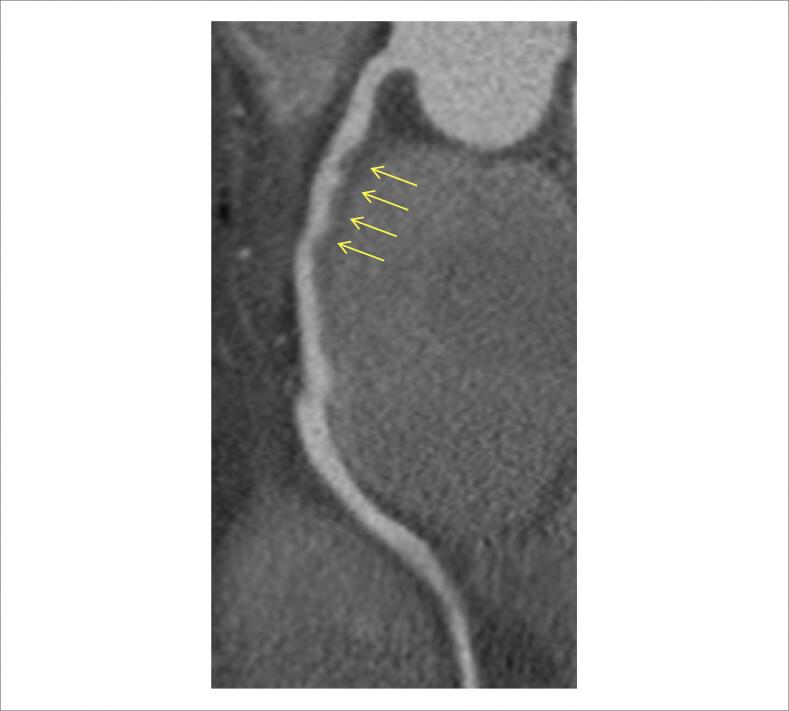
Angiotomografia coronariana de acompanhamento: Angiotomografia coronariana apresentando imagens sugestivas de trombos residuais (setas) em terço proximal-médio da coronária direita, com discreta redução luminal e leito distal pérvio.

O paciente recebeu alta hospitalar após quatro dias de internação, com alívio total da angina, em uso de AAS, apixabana e enalapril. Após 15 dias, permaneceu assintomático. Foi submetido a angiotomografia computadorizada de coronária que evidenciou trombo residual em terço médio da artéria coronária direita com discreta redução luminal e leito distal pérvio (Figuras 3 e 4).

**Figura 4:**
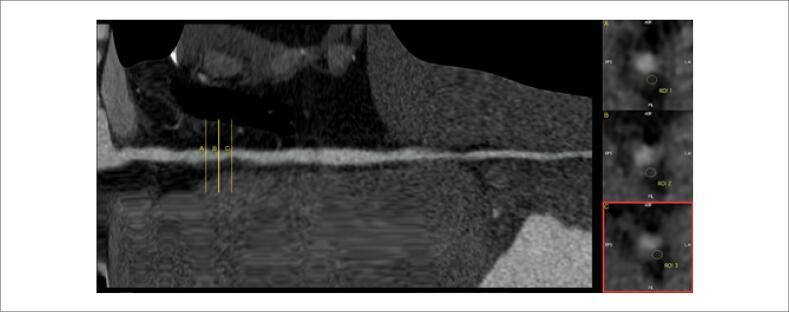
Angiotomografia coronariana de acompanhamento: Artéria coronária direita com estreitamento luminal irregular e segmentar de grau discreto em terço proximal-médio. Na visão transversal do vaso nas áreas de estreitamento, podem ser observadas imagens com baixa atenuação (20 a 100 HU), intimamente relacionadas à parede do vaso, que podem ser compatíveis com a hipótese de trombos.

## Discussão

A infecção pelo coronavírus SARS-CoV-2 foi declarada um evento pandêmico em março de 2020 pela Organização Mundial da Saúde (OMS), sendo responsável por elevada morbimortalidade em praticamente todos os países e territórios do mundo. Quando sintomática, a infecção apresenta-se mais comumente através de sintomas dos sistemas respiratório ou gastrintestinal, podendo também estar associada a manifestações cardiovasculares, desde lesão miocárdica até infarto agudo do miocárdio, miocardite fulminante e choque cardiogênico, aumentando a morbimortalidade da doença.^1^

Estudos anteriores mostraram que pacientes com covid-19 estão predispostos a eventos trombembólicos, tanto venosos quanto arteriais, com trombembolismo periférico e pulmonar, acidente vascular encefálico (AVE), infarto agudo do miocárdio e isquemia aguda de membros inferiores.^2^^–^^4^

O paciente descrito é um jovem sem fatores de risco para doença arterial coronariana, que apresentou episódio de IAM CSST inferior com alta carga trombótica, sem evidências de doença aterosclerótica em outras artérias coronárias e com pesquisa de RT-PCR positiva para covid-19. Por se tratar de um paciente sem outros fatores de risco conhecidos para trombose coronariana, é provável que a infecção viral e a resposta inflamatória sejam as protagonistas na ativação da cascata de coagulação como causa da trombose coronariana com manifestação clínica de infarto agudo do miocárdio.

Seif et al,^5^ Dominguez-Erquicia et al.,^6^ e Al-Sadawi et al.,^7^ descreveram casos de pacientes sem fatores de risco para doença arterial coronariana (DAC) que apresentavam IAMCSST e angiografia coronária que apresentavam trombo maciço com oclusão coronária sem doença aterosclerótica associada. O fato de serem pacientes sem fatores de risco para DAC e que não apresentavam placas ateroscleróticas coronarianas levanta a possibilidade de o evento trombótico estar associado ao estado de hipercoagulabilidade da infecção por covid-19. Nestes e no caso descrito por Lacour et al.,^8^ a trombose coronariana não foi associada à síndrome respiratória aguda grave, reforçando a possibilidade de eventos trombóticos mesmo em pacientes sem manifestações respiratórias ou sistêmicas graves.

Semelhante às ocorrências relatadas anteriormente,^5^^,^^7^ o caso a seguir destaca um paciente com covid-19 e IAMCSST, e que apresenta alta carga trombótica na angiografia coronária e ausência de critérios de reperfusão após terapia fibrinolítica, o que revela a necessidade de terapia de intervenção percutânea de resgate precoce. A grande quantidade de trombo deve estimular o uso de terapia farmacológica mais agressiva, como fibrinolíticos, inibidor da glicoproteína IIb/IIIa e uso prolongado de anticoagulantes. A ministração destes últimos, associada à terapia antiplaquetária por algumas semanas após o evento deve ser considerada, devido ao estado pró-trombótico associado à infecção por covid-19.

Em tal caso, o uso da angiotomografia coronariana para acompanhamento da lesão reforça a possibilidade de estudo coronariano não invasivo, o que permitirá a avaliação da placa além da luminografia. Ademais, no contexto da pandemia causada pela covid-19, a realização da angiotomografia reduz a exposição e os riscos para a equipe de saúde, permitindo, quando necessário, estudar alterações pulmonares em conjunto com a avaliação coronariana.^8^

## Conclusão

O infarto agudo do miocárdio com trombose coronariana é uma entidade que pode ser associada à covid-19 devido ao estado pró-trombótico predisposto pela infecção, mesmo em pacientes sem fatores de risco cardiovasculares sabidos. Nesses casos, tendo em vista a alta carga trombótica, uma terapia farmacológica agressiva, substituindo a angioplastia, deve ser considerada.
