# Supplementary material for: Infarto Agudo do Miocárdio com Trombose Coronária em um Paciente com Covid-19 sem Fatores de Risco para Doença Cardiovascular
Source: Arq Bras Cardiol. 2021 Mar 3;116(3):511–5. [Article in Portuguese] doi: 10.36660/abc.20200972 (PMC8159547; doi:10.36660/abc.20200972)
Supplement: Translation

# Translation: Acute Myocardial Infarction with Coronary Thrombosis in a Covid-19 Patient without Risk Factors for Cardiovascular Disease

**Authors:** Tainá Viana, Mariana Lins Baptista Guedes Bezerra, Rodrigo Morel Vieira de Melo, Cristiano Guedes Bezerra, Vítor Mamédio, Gabriela Pio Dourado, Clara Salles Figueiredo, Luiz Carlos Santana Passos

**Affiliations:** 1 SalvadorBA Brazil Universidade Federal da Bahia, Salvador, BA - Brazil; 2 SalvadorBA Brazil Hospital Ana Nery, Salvador, BA - Brazil

**Keywords:** Myocardial Infarction, COVID-19, Betacoronavirus, Young Adult, Coronary Thrombosis, Thrombolytic Therapy

## Case Report

A 32-year-old young man, without cardiovascular risk factors, sought an Emergency Unit due to severe chest pain 30 minutes before admission, without irradiation. Admission vital signs: T 36.1°C, heart rate of 89 bpm and peripheral O_2_ saturation of 96% in room air. During systematic interrogation, anosmia and ageusia were reported two days ago and fever or any other respiratory symptom was denied.

He was previously healthy and denied use of illicit drugs or previous history of angina. He denies a family history of acute myocardial infarction or coronary artery disease.

The 12-lead electrocardiogram (EKG) showed ST segment elevation in DII, DIII and aVF; and depression in DI and aVL, compatible with acute myocardial infarction with ST-segment elevation (STEMI) of the inferior wall (Figure 1) and positive troponin. Other laboratory tests showed changes in C-reactive protein of 6.7 mg/L (VR: <10mg/L), ferritin 350.2 ng/mL (VR: 21.8 to 274.6 ng/mL), LDH 5600 U/L (VR: 120 to 246 U/L) and leukocytosis of 12,450 cell/uL. Ultra sensitive troponin was above 50 ng/mL (VR: <0.034 ng/mL).

**Figure 1:** Eletrocardiogram of the admission. Electrocardiogram revealed ST segment elevation in DII, DIII and aVF and ST-segment depression in DI and aVL, compatible with inferior acute myocardial infarction.

The patient received an attack dose of dual antiplatelet therapy (aspirin 300 mg and clopidogrel 300 mg). As referral to angioplasty in less than 120 minutes would not be possible, fibrinolytic therapy was used, using Tenecteplase (delta T of 5 hours and 36 minutes). The post-thrombolysis EKG showed a 50% reduction in elevation of the ST segment, but the patient persisted with chest pain. Considered a failure of reperfusion, the patient was referred for rescue angioplasty. Cineangiocoronariography (Figure 2; video 1 and 2) revealed a right coronary artery with a large burden of thrombus in the middle and distal segment, without obstructive atherosclerotic lesions in other coronaries. Due to the high thrombotic load, despite the reduced flow (TIMI 2), it was decided not to intervene and to use dual antiplatelet therapy associated with low molecular weight heparin in a therapeutic dose for 72 hours. The patient was transferred to the intensive care unit with complete symptom relief.

**Figure 2:** Coronary angiography. (A) Right coronary with a large BURDEN of thrombus in its medial and (B) distal portions. (C) Left coronary artery without atherosclerotic lesions.

**Video 1:** Angiography of the right coronary. The coronary angiography revealed a right coronary artery with a large burden of thrombus in extensive mid and distal segment.

*Link: http://abccardiol.org/supplementary-material/2021/11603/2020-0972-Video01.mp4*

**Video 2:** Left coronary angiography. Coronary angiography without evidence of atherosclerotic obstructive lesion in the other coronary arteries.

*Link: http://abccardiol.org/supplementary-material/2021/11603/2020-0972-Video02.mp4*

COVID-19 was suspected, a nasopharyngeal swab was performed using RT-PCR for SARS-COV2, which was positive. Computed tomography of the chest, without changes. No specific therapy was instituted for COVID-19, as the patient remained without respiratory symptoms.

The patient was discharged after four days, with total relief of angina, using aspirin, apixaban and enalapril. After fifteen days, he remained asymptomatic and underwent coronary computed tomography angiography that showed a residual thrombus in the middle third of the right coronary artery with a slight luminal reduction and a patent distal bed (Figures 3 and 4).

**Figure 3:** Follow-up coronary angiotomography. Coronary angiotomography showing images suggestive of residual thrombi (arrows) in the proximal-middle third of the right coronary, with slight luminal reduction and a patent distal bed.

**Figure 4:** Follow-up coronary angiotomography. Right Coronary Artery with irregular and segmental luminal narrowing of a slight degree in the proximal-middle third. In the transversal view of the vessel in the narrowing areas, images with low attenuation (20 to 100HU) are closely related to the vessel wall, which may be compatible with the hypothesis of thrombi.

## Discussion

The COVID-19 infection was declared a pandemic by the World Health Organization in March 2020, being responsible for high morbidity and mortality in several countries around the world. When symptomatic, the infection most commonly presents with symptoms of the respiratory or gastrointestinal tract, which may be associated with cardiovascular manifestations, from myocardial injury to acute myocardial infarction, fulminant myocarditis and cardiogenic shock, increasing the morbidity and mortality of the disease.^1^

Previous studies have shown that patients with COVID-19 are predisposed to thromboembolic events, both venous and arterial, including peripheral and pulmonary thromboembolism, stroke, acute myocardial infarction and acute lower limb ischemia.^2^^–^^4^

The patient described is a young man with no risk factors for coronary artery disease, who had an episode of inferior STEMI with a high thrombotic burden without evidence of atherosclerotic disease in other coronary arteries and with positive RT-PCR for COVID-19. As he is a patient without other known risk factors for coronary thrombosis, it is likely that viral infection and the inflammatory response are the protagonists in the activation of the coagulation cascade as the cause of coronary thrombosis with clinical manifestation of acute myocardial infarction.

Seif et al.,^5^ Dominguez-Erquicia et al.^6^ and Al-Sadawi et al.^7^ described cases of patients without risk factors for coronary artery disease (CAD) who had STEMI and coronary angiography showing massive thrombus with occlusion coronary artery disease without associated atherosclerotic disease. Since these are patients without risk factors for CAD and without coronary atherosclerotic plaques, raises the possibility that the thrombotic event is associated with the hypercoagulable state of COVID-19 infection. In these and in the case described by Lacour et al.,^8^ coronary thrombosis was not associated with severe acute respiratory syndrome, reinforcing the possibility of thrombotic events even in patients without severe respiratory or systemic manifestations.

Similar to previously reported cases,^5^^,^^7^ this case describes a patient with COVID-19 and STEMI presenting high thrombotic burden on coronary angiography and absence of reperfusion criteria after fibrinolytic therapy, revealing the need for early rescue percutaneous intervention therapy. The large burden of thrombus should encourage the use of more aggressive pharmacological therapy, such as fibrinolytics, glycoprotein IIb/IIIa inhibitor and prolonged use of anticoagulants. The use of anticoagulant associated with antiplatelet therapy for a few weeks after the event should be considered due to the prothrombotic state associated with COVID-19 infection.

The use of coronary angiotomography to monitor the lesion in this case reinforces the possibility of a non-invasive coronary study allowing the evaluation of the plaque in addition to luminography. In addition, in the context of the COVID-19 pandemic, the performance of angiotomography reduces exposure and risks for the health team, allowing, when necessary, assessment of pulmonary changes in conjunction with coronary assessment.^8^

## Conclusion

Acute myocardial infarction with coronary thrombosis is an entity that can be associated with COVID-19 due to the prothrombotic state predisposed by the infection, even in patients without known cardiovascular risk factors. In these cases, in view of the high thrombotic burden, aggressive pharmacological therapy should be considered, instead of angioplasty.
